# AI-based healthcare: a new dawn or apartheid revisited?

**DOI:** 10.1007/s00146-020-01120-w

**Published:** 2020-12-22

**Authors:** Alice Parfett, Stuart Townley, Kristofer Allerfeldt

**Affiliations:** 1 Integrated PhD student at the Centre of Doctoral Training for Accountable, Responsible and Transparent AI, University of Bath, Claverton Down, Bath, BA2 7AY UK; 2 BSc from the University of Exeter, Penryn Campus, Penryn,, TR10 9FE Cornwall UK; 3 University of Exeter (Environment and Sustainability Institute), Penryn, Cornwall UK; 4 University of Exeter (Humanities), Penryn, Cornwall UK

**Keywords:** Artificial intelligence, Healthcare, Bias, History, Mathematics

## Abstract

The Bubonic Plague outbreak that wormed its way through San Francisco’s Chinatown in 1900 tells a story of prejudice guiding health policy, resulting in enormous suffering for much of its Chinese population. This article seeks to discuss the potential for hidden “prejudice” should Artificial Intelligence (AI) gain a dominant foothold in healthcare systems. Using a toy model, this piece explores potential future outcomes, should AI continue to develop without bound. Where potential dangers may lurk will be discussed, so that the full benefits AI has to offer can be reaped whilst avoiding the pitfalls. The model is produced using the computer programming language MATLAB and offers visual representations of potential outcomes. Interwoven with these potential outcomes are numerous historical models for problems caused by prejudice and recent issues in AI systems, from police prediction and facial recognition software to recruitment tools. Therefore, this research’s novel angle, of using historical precedents to model and discuss potential futures, offers a unique contribution.

**Supplementary Information:**

The online version contains supplementary material available at 10.1007/s00146-020-01120-w.

## Introduction

Wong Chut King, a Chinese labourer, was found dead in the basement of a hotel in San Francisco’s Chinatown in March 1900 (Risse 2012). After an ‘egg-shaped blackish lump’ was discovered in his groin, King would prove to be the first confirmed victim of San Francisco’s Bubonic Plague outbreak at the turn of the twentieth century (Risse 2012: 76). Unbeknownst to most at the time, King’s death would instigate a public health and political crisis, with infighting tearing the city apart and an intensification of Sinophobia (Skubik 2002).

At the end of the nineteenth century, San Francisco was home to California’s largest Chinese population and traditional Gold Rush arguments remained: Chinatown was filthy, and its bad smells bred disease (Risse 2012). Germ theory was in its infancy and the plague was considered an “Oriental disease” (Morton Todd 1909). This scapegoating was not new; Malaria, Smallpox and Leprosy were all placed at their door (Trauner 1978). Racial prejudices justified such claims; the phrase ‘yellow peril’, coined at the time, demonstrated the supposed threat that Asian immigrants represented for European-American civilisation (Risse 2012). In the 1870s, these racist arguments broadened as Chinatown grew. It was argued that their cheap labour undermined wage rates, their poor sanitation endangered the nation and they were ‘inferior in organic structure, in vital force, and in the constitutional conditions of full development’ (Trauner 1978: 70–72). The result was the Chinese Exclusion Act of 1882, which dictated policy for the next sixty years. Popular racism enabled the maltreatment of the Chinese and explains how politicians could disregard the plague in Chinatown.

Multiple quarantines were enacted over the period, along with a proposed vaccination programme (Skubik 2002). The vaccine used dead bacteria but rumours that it was poisonous left Chinatown on the verge of riot (Skubik 2002). Meanwhile, California’s Governor, along with San Francisco’s business community, chose to deny the existence of the plague and suppress facts to protect their interests (Skubik 2002). Many anti-Chinese groups considered using the plague as an excuse to destroy Chinatown (Risse 2012). As one scurrilous commentator put it: “The only way to get rid of that menace [plague] is to eradicate Chinatown from the city… clear the foul spot from San Francisco and give the debris to the flames” (The San Francisco Call 1900). This was arguably only ruled out due to fears over disrupting trading links with China (Risse 2012).

In 1900, the disease was mainly spreading among the rodent population (Skubik 2002). Real action was only taken in 1903, once the disease had become highly contagious and fatal and with a new Governor in office (Skubik 2002). The epidemic was declared over in summer 1904 after 122 deaths, most, but not all, of whom were Chinese (Skubik 2002). The lack of genuine medical help offered, compared to the speed with which Chinatown was blamed, is symbolic of the period’s prejudices. Those dealing with the epidemic were driven by their own interests; only when the impact was felt outside Chinatown, was action taken.

So, how is this tale from history relevant to the rapid growth of Artificial Intelligence (AI) today? The connection is bias. In San Francisco in 1900, prejudice was present in multiple forms, demonstrated by numerous characters and its impact had life and death consequences for those on the receiving end. Also, this case study offers the first example, of many for this piece, of how such prejudice can trigger a negative feedback loop. That is, as the plague worsened, Sinophobia intensified, further limiting the response and allowing the loop to repeat. Unfortunately, over 100 years on, similar biases and feedback loops are emerging, only this time our technological creations have the potential to be biased. On various scales and in areas from recruitment to policing, bias could emerge and if unchecked could guide decisions worldwide.

This argument for the existence of potential ‘bias’ in AI systems is not without foundation. Humanity’s capacity for prejudice in society, our thinking or actions is evident throughout history, whether that bias be unconscious or deliberate. O’Neil (2016: 203–204) takes this further, suggesting that ‘injustice, whether based in greed or prejudice [or both], has been with us forever’. O’Neil acknowledges that human decision making can often be flawed but can also evolve, unlike automated systems which ‘stay stuck in time until engineers dive in to change them’ (O’Neil 2016: 203–204). This inference that humanity’s biases can sometimes be replicated in our technological creations also appears in Whitaker, Colombo and Rand’s (2018: 10) findings: ‘the distributed collective intelligence of machines is also a social endeavour, and it is potentially susceptible to prejudicial phenomena as seen in the human population’. Sismondo (2010: 9) agrees regarding this social side of technology, arguing that ‘people act in the context of available technology’.

So, given this idea that technology influences social conduct, which in turn determines the technology humanity uses; it seems unlikely that even the most sophisticated AI-based technologies will be able to avoid the ‘forever’ phenomenon that is bias. Yet, as Plant (1997: 46) states, resources that ‘were once restricted to those with the right face, accent, race, sex’ are now available to all. With that in mind, this paper works to draw attention to various case studies where the potential for bias has become a reality and has served to subvert AI’s beneficial potential.

This paper chooses to delve deeper into this wider suggestion of AI’s ability to embody various biases. To do this, it will focus on an area of the most pressing importance should such biases emerge: healthcare. Specifically, a toy model imagining an AI in control of a vaccination programme in the event of an epidemic will form the basis of this article. From this basis, the paper has an overarching narrative which is split into three sections (called stories) for structural reasons. While the scenarios discussed are strongly intertwined, they are tackled separately here to demonstrate the multiplicity of the dangers. The first proposes that this fear of the algorithm is an overreaction. It will proffer that the outcomes can be the same, no matter if the algorithm is altered, hence, there is nothing to worry about? In contrast, the second story observes how seemingly insignificant changes to the code and alterations to the datasets used can hugely alter the outcomes for individuals. Finally, the third story alters the dataset again to demonstrate the consequences when supposedly ‘clear’ instructions are written into AI systems and yet when the programme is allowed to run freely, deviation from these instructions can occur. Then, a discussion will draw together all these ideas from the toy model and reference real-life situations, including referring back to the example of San Francisco’s Chinatown, to further enhance this paper’s arguments.

The toy stochastic network modelling approach used here is sufficient to highlight the issues raised within the scope of the paper, where the focus is on the interactions and feedbacks between data, different actions and potential outcomes. The target of this paper is not to offer novel AI tools for the healthcare, but rather to draw attention to and stir a debate regarding the potential outcomes, should the feedbacks discussed here become a reality in AI systems. It is appreciated that in the ‘AI in Healthcare’ context in reality, there are many more complex models already in the field. A deeper and more comprehensive investigation into the intricate and sensitive topic of AI in healthcare scenarios in the ‘real-world’ would draw on a wide range of models. For example, Brailsford et al. (2009) analysed frequency of use, domains of application and the level of implementation of a wide range of research modelling approaches in the healthcare remit. In fact, they note that ‘simulation methods are prominent in planning and system/resource utilisation’ which enhances the legitimacy of this paper’s choice of modelling technique to effectively meet its target (Brailsford et al. 2009: 139). Brailsford et al. (2009) demonstrate the sheer range of potential methods to be used but regarding the earlier suggestion of more complex alternative models, Weng et al. (2017) offer an excellent overview of some of the machine-learning models being used to help improve cardiovascular risk prediction. They looked at four different machine learning algorithms compared to an established algorithm that predicts future risk based on well-established factors such as cholesterol or smoking (Weng et al. 2017). On the other hand, the machine learning approaches can exploit ‘big data’ and the neural network model performed the best in their study (Weng et al. 2017). Therefore, the two mentioned studies demonstrate the wider ranging and more complex nature of models currently at work in the ‘AI in healthcare’ field. However, they also evidence that for the scope of this paper, the simple stochastic network models used are ideal as suggestions of what could happen, and the use of features—even on this paper’s smaller scale—is clearly a well-founded technique in this field.

## Methodology

Skubik’s work on the bubonic plague in San Francisco’s Chinatown, in particular, highlights that it was assumed at the time that only Chinese people required vaccination for this ‘oriental disease’ (Skubik 2002). Hence, it can be assumed that assigning an experimental, and potentially dangerous, medicine was based on prejudice. This work will take this suggestion of bias guiding healthcare decisions further, by imagining that issuing vaccinations fell to an AI system. This paper will examine how that might be decided and look into some of the implications of that automation.

**Fig. 1:**
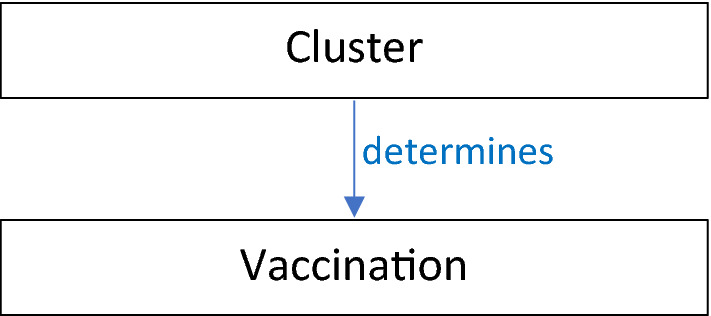
Diagram simplifying how recipients of the flu vaccine are determined, for example. The cluster that people are slotted into, according to age, determines whether they receive the vaccine

Age is often used to determine who receives a vaccine. For example, over sixty-fives are one of the groups specified for the free flu vaccine in the UK (NHS UK 2019).^1^ Similarly, Gupta et al. (2014) state that ‘top-ranked influential spreaders need to be identified for targeted immunization’ and that traditionally ‘nodes with high centrality are considered to be the influential nodes’. Although they admit that many measures can be used for this ‘centrality’ parameter, they note that ‘degree centrality’, where each node is given an ‘importance score based simply on the number of links held by each node’ (Disney 2020), is a popular choice (Gupta et al. 2014). More generally, in any network or population of people, every individual has a vast number of different attributes, including age and weight but also race and religion, or any number of medical conditions. These attributes could be linked to susceptibility and, like age for the flu vaccine, be used to classify or, in AI parlance, cluster individuals for the purposes of determining a vaccination policy Fig. 1. Although, populations are not an unstructured, homogeneous wholes in which disease spreads out. They are heterogenous, structured by social connections, be they physical or virtual via social media. For this toy model, two types of parameter will be used as metaphors for all the various attributes of individuals in a population. The first will be referred to as health parameters, which for this toy model will be summarised by age and weight. The second are network parameters, which will be simplified to the edge degree of each node in the population (number of connections an individual has to others in the population).

In large, high-dimensional networks of populations with multiple and varying datapoints describing individual features, determining clusters is beyond human action but well within the scope of powerful AI systems. Such AI-based decision systems are already in development in the healthcare context, particularly in the context of Acute Kidney Injury (Argyropoulos et al. 2019). Google DeepMind’s initially developed a machine capable of playing the ancient Chinese game of Go (DeepMind 2019). Now, by use of a neural network, they are connecting around 9000 data-points and historic incidents of kidney injury for a dataset of American veterans in an attempt to predict acute kidney injury before it happens (Powles 2019). Admittedly, at present, the accuracy is only around fifty-five percent, but the example proves the extent to which social connections and features are constantly being captured, processed and used to make decisions (Powles 2019). If you doubt this statement, no one knew when their data had been sold to Cambridge Analytica and even now the scandal has been unearthed, it is unlikely that you know whether you were a victim (Amer and Noujaim 2019). So, how might this clustering and AI-based vaccination lead to biases in future healthcare?

### The Model

In an attempt to explore this potential relationship, a ‘toy model’ was created that uses networks to model a hypothetical disease. The health parameters—age and weight, and network parameter—social connections, of twenty-five people, were recorded to give a picture of potential features available to such systems. By social connection, this term was considered to imply a relationship through which a disease could be contracted and corresponds to the edge degree of each node. Of course, this is an idealised view of AI-based disease management. However, it does not detract from the essential point being made, that of the interactions and feedbacks between data, vaccination, and propagation of the disease. In fact, it is argued that such a ‘toy model’ better depicts these potential outcomes than if realism was considered a factor.

So, in this fictionalised model, what are the rules?

- The features (age, weight, and edge degree) of each person are used pair-wise to cluster the population and the disease spreads through the population via the social connections outlined.
- Individuals are assigned a susceptibility to the disease according to their features, a value that reduces should they be assigned the vaccine.
- Inherently, a person with more connections to those infected/vaccinated in the network will have a higher/lower probability of retaining the infection/recovering.
- Once the vaccination is inputted into the simulation, the vaccine is applied at each timestep provided that the person remains in the vaccinated group.
- Individuals can recover from the disease in this idealised model.
- Only nodes (individuals) that have not been ill for a lengthy period are used for clustering purposes. This is used to represent how app-based data collection will depend on the app being active.

**Fig. 2:**
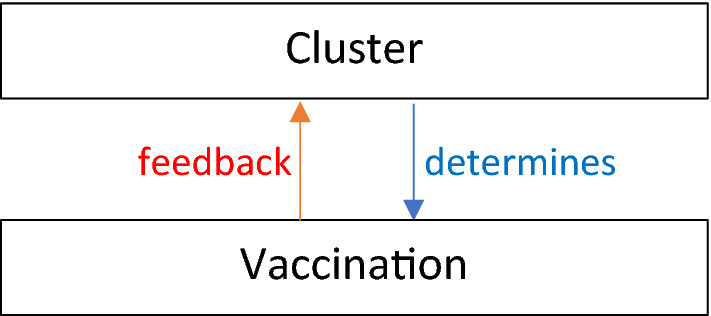
Diagram simplifying how recipients of the vaccine in this work's model are determined. As before, clustering determines vaccination receipt but this time, vaccination, i.e. whether the person is well enough to be considered, feedbacks on the clustering process, if they are not well enough, they will be excluded from the clustering process

At the outset of this model, the clustering process is the only element in action. Then, to replicate the situation detailed in Fig. 2, the exclusion element of the model commences and is iterative and repeats after every timestep. The point being made here is that AI systems can continuously re-classify features. So that if, for some reason, data are omitted for a node, then this will impact or feedback, on clustering. Consequently, as the disease outbreak progresses so potential biases may emerge due to this feedback between the data, vaccination, and disease impact. For example, apps that collect exercise data would miss data for individuals who did not exercise because of illness. But that missing data would influence cluster membership, vaccination and, in turn, recovery from illness. Similarly, immigrants without papers or modern slaves would have no medical records or online presence. Therefore, they would not just be missing some data, they would have none and under this system of vaccination, they would always be excluded from vaccination. In the simulations, people who have been infected for a long time are excluded from clustering to illustrate the impact of this feedback on clustering. Whilst this model’s outcomes may be simpler than reality, there are numerous parameters in Table 1, some set and some chosen by the modeller, that can alter these outcomes.

**Table 1:** Itemised list of all the parameters required for this model, including the inputted values and the variable used to represent each parameter in the code

Parameters chosen by the modeller		
Number of cluster groups	nocl	4
Susceptibility of vaccinated nodes	susl	0.1
Susceptibility of unvaccinated medium to low	susml	0.2
Susceptibility of unvaccinated medium to high	susmh	0.6
Susceptibility of unvaccinated nodes	sush	0.85
Length of time	T, Q	The model runs for 100 timesteps 1000 times
Proportion of time a node has to be infected for before being excluded from the clustering	tolill	5 times in the last 10 timesteps
Initial number of those infected	y	Nodes 15 to 20
Which cluster group(s) is/are vaccinated – the union of those over the threshold for the two features used in certain plot	age_tol, weight_tol, degree_tol	60 for age, 65 for weight and 5 for degree
When the exclusion process starts in the model	T1	10
When the vaccination starts in the model	T2	20
*Set parameters*		
The adjacency matrix for the population	A	Appendix 1 in ESM
The features of the members of the population	feats	Appendix 1 in ESM

*Set parameters*		
The adjacency matrix for the population	A	Appendix 1 in ESM
The features of the members of the population	feats	Appendix 1 in ESM

This ‘toy model’ was produced in MATLAB (Appendix 2 in Electronic Supplementary Material). The model is built around an adjacency matrix (Appendix 1 in ESM), which depicts the connections in the population through which a disease could be contracted and is read into the programme, from an Excel spreadsheet, at the outset. At the same stage, the population’s features are also read into the model, (Appendix 1 in ESM). In fact, all the parameters noted in Tab. 1 are created before the iterative process begins in the model. Whether an individual becomes infected is a random process driven by the susceptibility probabilities. Therefore, the 100-timestep simulation is repeated 1000 times and the plots created in this work and the results discussed depict the summary statistics of all of these runs.

**Fig. 3:**
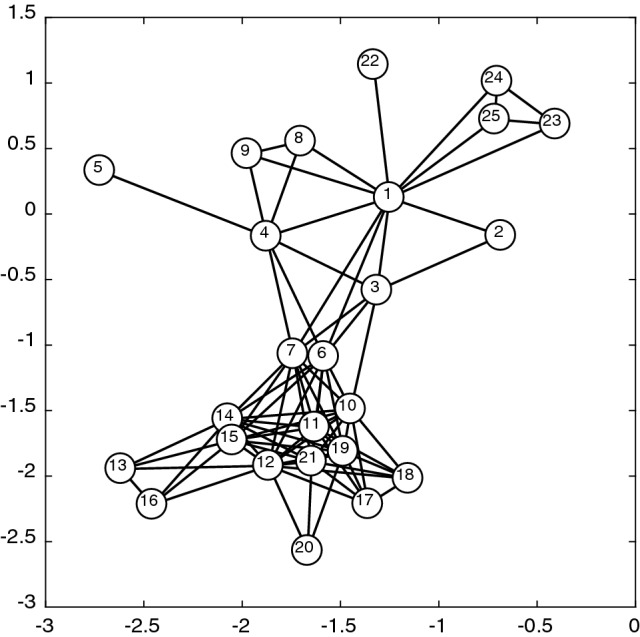
Network depicting the social connections between members of the population (before the disease/vaccination)

**Fig. 4:**
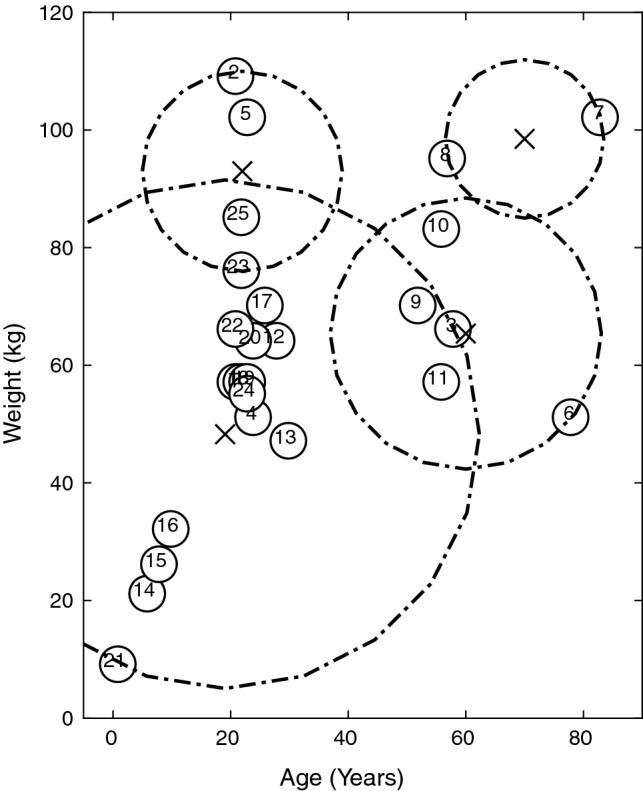
Plot depicting the members of the population according to their features (before the disease/vaccination)

Before the disease simulation is enacted, two corresponding plots Figs. 3 and 4 are produced to show the population originally (Appendix 3 in ESM). Note, the numbers on the plots correspond to the individuals in the population. The first, Fig. 3 is the network for the population, whereby the black edges correspond to the connections in the adjacency matrix, and the position of the nodes on the fixed axis is determined by the use of the fruc_rein function. This function uses the Fruchterman–Reingold Algorithm to find the optimal node placement for the network from the adjacency matrix, this is just one of many methods for node layout. Figure 4, plots the nodes according to their ages and weights. They are then clustered into four groups using kmeans clustering at this stage purely to demonstrate where these clusters lie before the disease or vaccination elements of the model. The centres of these clusters are shown by the crosses in Fig. 4 and their size is identified by the dash-dot rings.

**Fig. 5:**
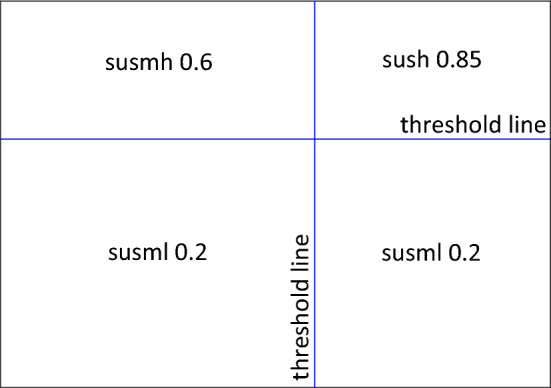
Susceptibility quadrants for the first and second stages of the simulation when only the clustering aspect is active, and the exclusion element commences

As detailed in Tab. 1, there are three stages to each simulation (Appendix 2 in ESM). The first lasts until timestep nine. Within this stage, the disease is permeating through the population and they are being constantly clustered into four groups based on their features (the pairs of either age and weight, age and degree, or weight and degree) by using kmeans clustering. The population’s susceptibilities to the disease depend on where the centre of their cluster falls in relation to the thresholds for each feature, detailed in Table 1. See Fig. 5 for these susceptibility quadrants during this first stages of the simulation.

During the second stage, the exclusion part of the model commences from timestep ten to timestep nineteen. As stated in Table 1, this means that individuals who have spent five or more of the last ten timesteps infected are no longer included in the clustering process. Note, if an individual has previously been excluded but they recover and spend time uninfected, they can be re-entered into the clustering process.

**Fig. 6:**
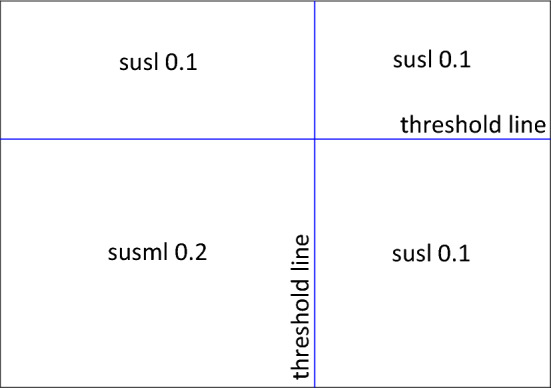
Susceptibility quadrants for the third stage of the simulation once the vaccination has started, hence lowering the susceptibilities for the union of the thresholds

**Fig. 7:**
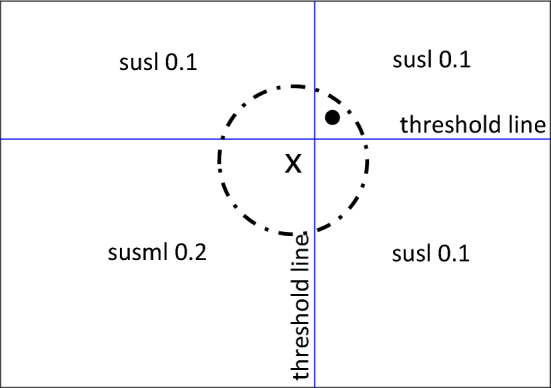
Example of how the usage of the cluster centres to determine vaccination can leave some individuals who are within the threshold without the vaccine

Finally, from timestep twenty and then for the remainder of the simulation, the vaccination process is included in the model. For those being vaccinated, their susceptibility to the disease is reduced to 0.1. Note, the exclusion process remains active. Hence, only a proportion, those being clustered, of the population are considered for the vaccination at any one timestep. Only nodes whose cluster group centre is in the union of the two axis thresholds, and are being clustered at that timestep, are in receipt of the vaccination. See Fig. 6 for the susceptibility quadrants for this final stage of the simulation and Fig. 7 to identify how the reliance on the cluster centres to determine vaccines impacts the susceptibilities.

Each of the stories that follow include a different pairing of the health and network parameters discussed previously. For each of these pairings, two simulations are run. The difference between the two depends on a variable called the ‘seed’, specifically an initial guess at the cluster centres. Simply, this parameter determines how the clustering simulation commences on each run. The first plots for each pairing use a fixed seed, meaning that for each of the 1000 runs of the model, the simulation will always start from the same clustering centres. On the other hand, the second plots use an updating seed which means that the model takes the previous run’s cluster centres as its new starting point or seed. This may only seem like an insignificant alteration but that is the reason for its inclusion; to demonstrate how such a small change could potentially impact the outcomes of the model hugely. Plainly, the outcome of the model could depend on the programmer on shift. To fix the seed when the rest of the model is constantly changing could be questioned. Equally, to keep changing elements of the model continuously, including even the starting point could be questioned. Both options can be justified and neither option is wrong or right. Hence, why this seed altering aspect of this paper is included, to demonstrate the sheer unpredictability of such small changes.

Four different plots are used throughout the three stories. The first plots the nodes according to their health and network features in question (Appendix 4 in ESM). Also, included on these plots are the cluster ellipses and centres to demonstrate the average size of the groups and average placement of the centres, represented by the dash-dot rings and crosses, respectively. As these plots are an average of the 1000 runs over the 100 timesteps, the plots include ‘shadows’ for both the ellipses and crosses. These shadows are the average of the 1000 runs at each specific timestep so they show how the groups have moved over time with the shade darkening as the simulation nears the end. The threshold lines are marked on these plots. The cluster centres must be above these threshold lines for the nodes included in their group to be considered for the vaccine, assuming they are still being clustered at that point in time. Finally, the radius of the nodes themselves demonstrates the probability that they were vaccinated at a given time. A larger ring around a node means a higher probability of being vaccinated. Shading of the ring indicates progression of time—from light to dark as simulation time progresses. Note, two of these style plots will be included in each section, corresponding to the fixed or updated seed alteration described above.

The second plot used is only included in story one, primarily to aid with the reading of the plots described above, particularly the node radius element. This plot includes twenty-five line plots, one for each individual in the population, detailing the probability of being vaccinated over the course of the simulation (Appendix 5 in ESM). Also, included on each plot is one standard deviation either side of the mean line. Hence, this plot aids understanding of how the overall simulation is impacting on vaccination of each member of the population.

Story two includes the third plot for this paper. Again, this plot includes twenty-five line plots, one for each individual in the population. However, this time, these plots show the probability of being infected over the course of the simulation (Appendix 6 in ESM). Again, one standard deviation either side of the mean line is included. These plots are included as story two focuses primarily on the fate of the individual; hence, it is easy to see exactly how each individual is affected and whether their probability of infection is increased by the seeding changes. Therefore, this plot is repeated for both types of seeding.

Finally, story three focuses on how the outcomes can completely change with a different pairing of features and the seeding element. Hence, the final plots used in this piece show the variance in both the centres and size of the cluster groups over the course of the simulation for both seeding options (Appendix 7 in ESM). These plots each include three line plots, one for the x-coordinate of the centres, one for the y-coordinate, and one for the radius of the clusters and there are lines for all of the cluster groups.

## Results

### Story one: Fear of the algorithm overreaction—How it could all turn out fine

So far, this paper has somewhat implied that this toy model will create negative feedback loops and negative outcomes. However, what if that is not the case? What if the model does what it is meant to do, vaccinate those cluster groups above the threshold and overall help the population deal with this fictional disease? Equally, what if the model also does not change dramatically when the seeding changes, the outcomes are essentially the same?

This first story shows just this scenario. The health parameter used is weight and the network parameter, edge degree.

**Fig. 8:**
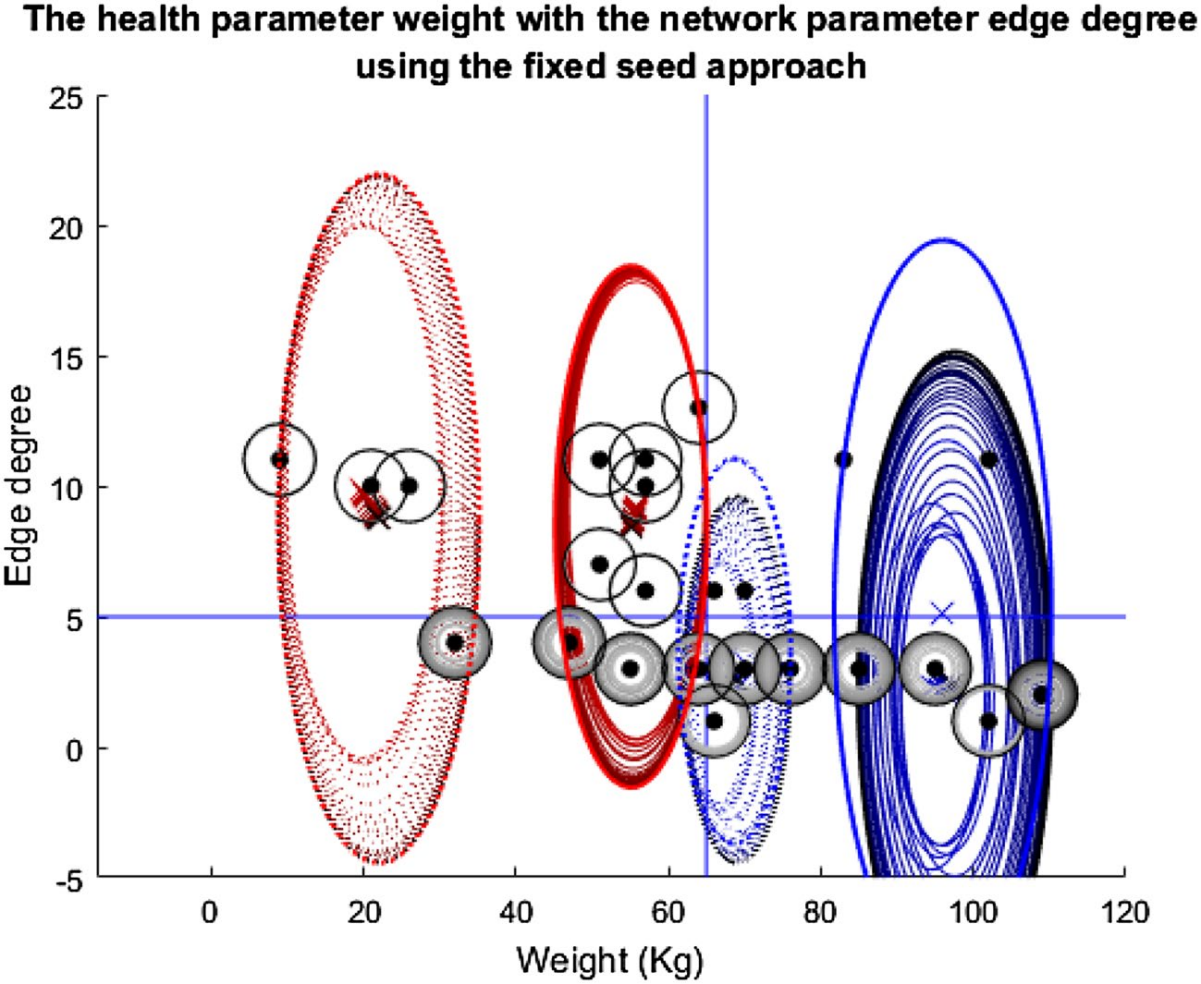
Plot depicting the population according to their edge degrees and weights using the fixed seeding. The four cluster groups are marked by the red and blue ellipses: Blue-dotted = Cluster 1; Red-dotted = Cluster 2; Blue-solid = Cluster 3; Red-solid = Cluster 4. The cluster groups at the start of the simulation are marked by the primary red and blue colouring, and the end point is marked by the black ellipses. The shades in between correspond to how these change over time. The same is true for the crosses which mark the centre of the clusters. The individual nodes are marked by the black dots. A black ring around these signifies that they are vaccinated all the time, the rings which go from white to black signify an increasing probability of vaccination and no circle at all means that they are never vaccinated. Finally, the blue lines at y = 5 and x = 65 mark the thresholds for vaccination

In Fig. 8, almost everyone at some point in time is in receipt of the vaccination and a large number receive it 100 percent of the time. All of the cluster groups collapse in size as the exclusion element is added into the model, but they are all able to recover steadily almost to their starting size as the vaccine is effectively distributed among the population, in turn reducing infection levels. Notably, the four nodes above both the threshold lines, therefore most in need of the vaccine, are unable to recover from the disease for long enough to be clustered, they are excluded throughout. This could be due to their location in the network and number of social connections meaning they are constantly being infected through contact with infected individuals.

**Fig. 9:**
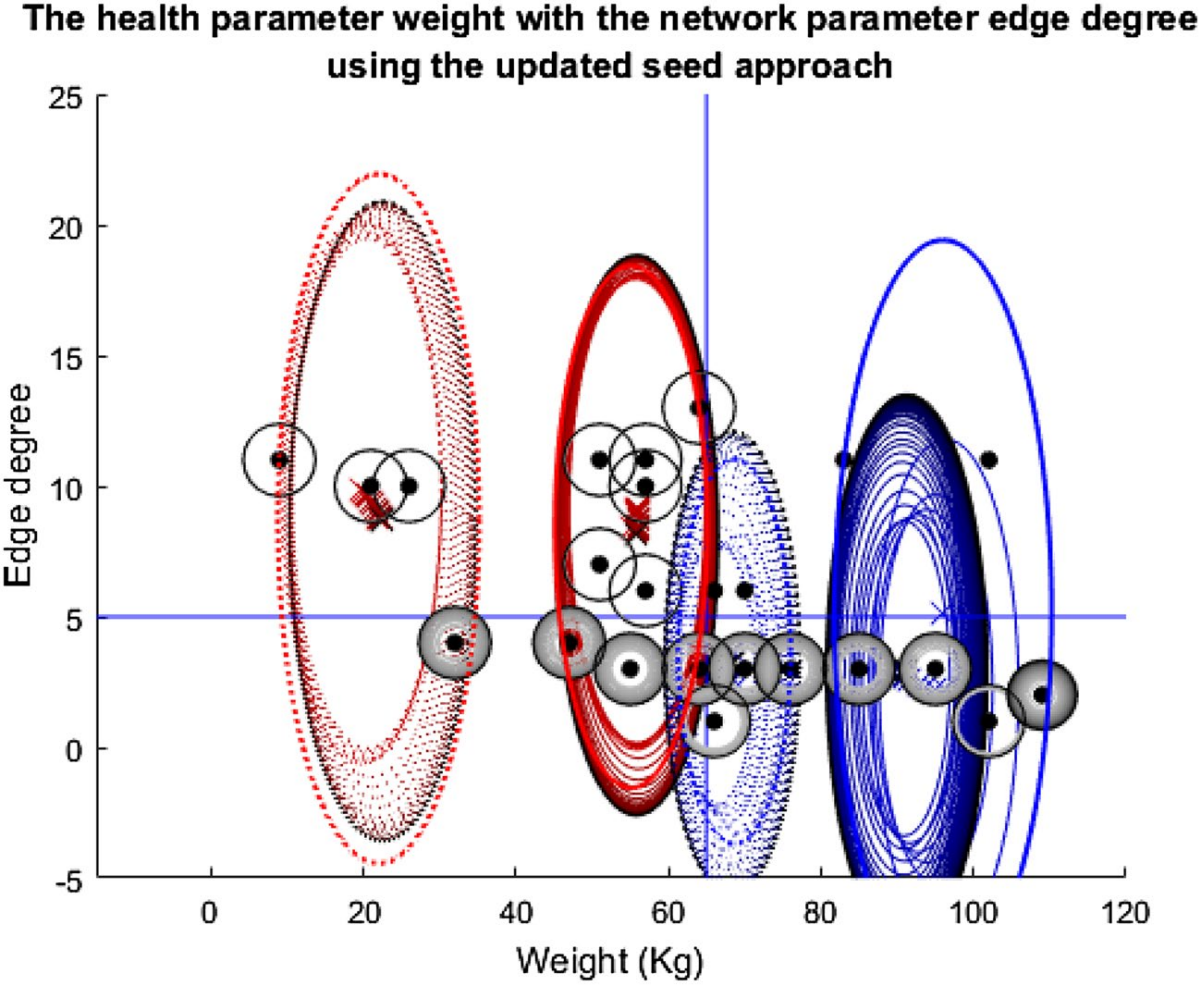
Plot depicting the population according to their edge degrees and weights using the updated seeding. For details on how to read the plot, see Fig. 8 caption

Figure 9 is almost identical to Fig. 8, despite the change in the seeding technique. The same nodes are excluded and the cluster group size recovery over time is very similar. The most obvious difference is the slight shift in the right-most blue-solid cluster group to the left in Fig. 9 compared to Fig. 8, but this seems to have minimal overall effect on the population and individual-level outcomes.

**Fig. 10:**
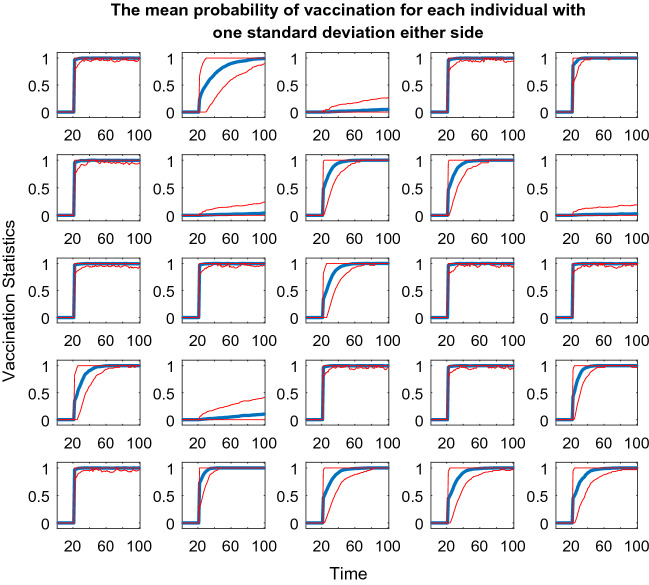
Twenty-five line plots showing each individual's average probability of receiving the vaccine at each timestep during the weight vs edge degree updating seed simulation. The blue central line marks this mean result. The two red lines either side are one standard deviation from this mean to each respective side. As this plot regards probabilities, the y-axis has been capped at zero and one

Figure 10 helps with the reading of the other plots in this paper as it adds context to the rings around the individual nodes in the above and following plots. As discussed for Fig. 9 many of the nodes are in receipt of the vaccination, as seen by how many of the line graphs in Fig. 10 are at one on the y-axis. Hence, Fig. 10 makes it easy to see how the vaccination programme is successful for the population as a whole. However, equally obvious are the four nodes that never receive the vaccine, are often infected and are excluded from the clustering at an early stage.

Overall, from the plots in this story, it would be easy to conclude that the clustering, exclusion process and alteration with the seed make very little difference to the outcomes of the model for the population as a whole and for individuals. Therefore, it would be easy to stop here and conclude that the algorithm has completed its task successfully and that this idea of negative feedback loops is disproven. However, one experiment does not reveal a pattern, it is important to follow the other stories and observe how the other feature pairings affect the simulation’s outcomes.

### Story two: Impact of a small change and dataset choice—How a seemingly insignificant alteration can change everything for the individual

Story one has shown just one of this toy model’s outcomes by using only one of the potential pairings of features. Now, story two uses a new pairing, the health parameter age with network edge degree. It is vital to appreciate the importance of such dataset choices and small changes, such as the seeding alteration, and the affect these can have on individuals. As already discussed, populations can have countless features; hence, unconscious or conscious biases emerging in AI systems have the potential to impact a wide range of different people, since they can target any of these features. Rittmuller (2018) claims that ‘minorities have much to fear from an AI future’, which is difficult to argue against. However, there is another point that is important to comprehend; these groups that are excluded or highlighted by these systems are not homogeneous wholes. Within each group are individuals and the decisions made by these AI systems could have life-changing impacts on these people.

**Fig. 11:**
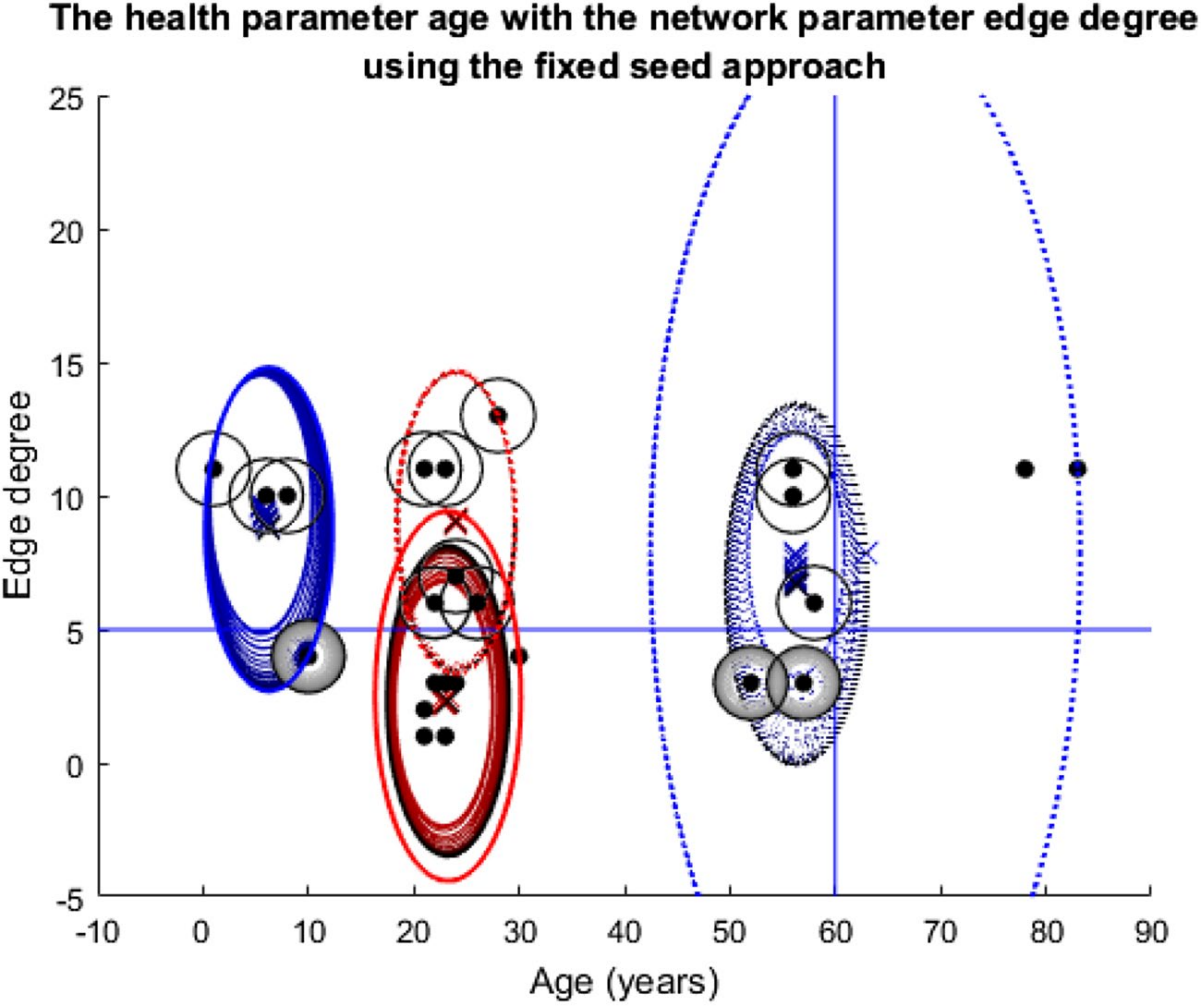
Plot depicting the population according to their edge degrees and ages using the fixed seeding. The blue lines at y = 5 and x = 60 mark the thresholds for vaccination. For details on how to read the plot, see Fig. 8 caption

Compared to Figs. 8 and 9, the different outcomes caused by the change in dataset in Fig. 11 are clear. The left blue cluster and the two red clusters do collapse a little and recover. However, the blue-dotted cluster collapses completely and fails to recover which leaves the two nodes above both thresholds completely excluded and unvaccinated for the duration of the model. Below the degree threshold line, there is a whole group of nodes that suffer a similar fate, very different to the simulations in story one where almost all the nodes at some point received the vaccine.

**Fig. 12:**
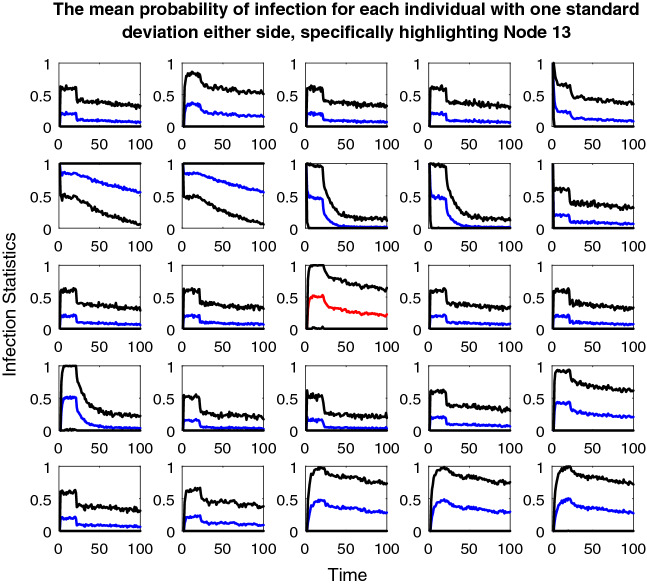
Twenty-five line plots showing each individual's average probability of being infected with the disease at each timestep during the age vs edge degree fixed seed simulation. The blue central line marks this mean result, except for the plot for Node 13 which is red (the reason for this highlighting follows Figs. 13 and 14). The two black lines either side are one standard deviation from this mean to each respective side. As this plot regards probabilities, the y-axis has been capped at zero and one

From Fig. 12, the impact on each individual in the population during the simulation in Fig. 11 is clear. The majority of the nodes seem to hover around the fifty percent chance of being infected mark, which demonstrates how by using this dataset, the population fairs much worse than the simulations in story one, whereby almost all the nodes received the vaccine so would have had a much lower probability of being infected on average.

**Fig. 13:**
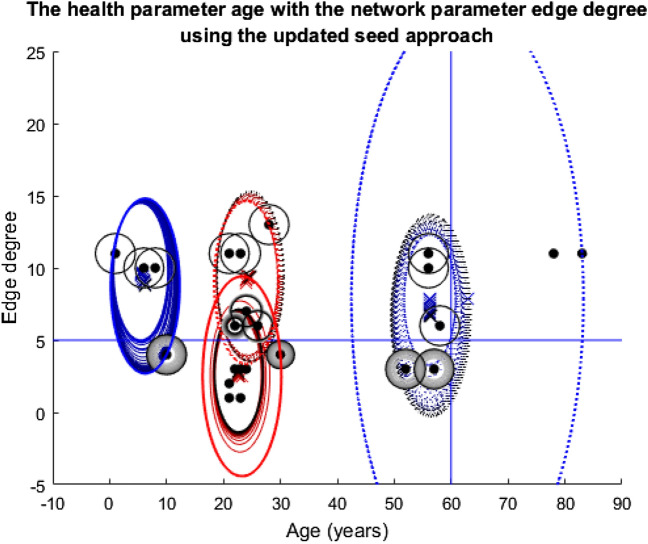
Plot depicting the population according to their edge degrees and ages using the updated seeding. The blue lines at y = 5 and x = 60 mark the thresholds for vaccination. For details on how to read the plot, see Fig. 8 caption

At a population-wide level, Fig. 13 seems very similar to Fig. 11. The collapsing and recovery process of the cluster groups is almost identical. Also, the nodes that are excluded from the clustering and unvaccinated throughout on first look seem to be the same. However, this is where Node 13 (Age = thirty, Edge degree = four, node that meets the solid red cluster group starting line, situated just below the degree threshold line) is very important. In Fig. 13, this node has a darkening black ring around it, which as previously described means that its chance of vaccine increases over the simulation. However, in Fig. 11, this very same node is excluded for the duration of the model and is never vaccinated.

**Fig. 14:**
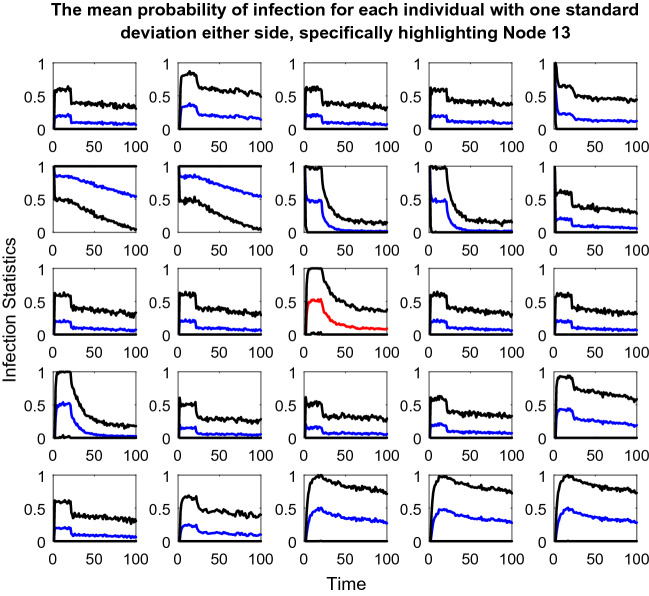
Twenty-five line plots showing each individual's average probability of being infected with the disease at each timestep during the age vs edge degree updated seed simulation. For details on how to read the plot, see Fig. 12 caption

Figure 14 further highlights the difference in fate for Node 13 when compared to Fig. 12; hence, this node’s red line compared with blue, in order to draw attention to the change. Compared to Fig. 12, all the other line plots in Fig. 14 seem almost identical, just as Fig. 11 and Fig. 13′s plots seemed almost identical. For Node 13 however, the change in approach to seeding has meant a change from a probability of being infected of approximately 0.3 down to 0.1, so a twenty percent difference, which in the context of contracting a disease is dramatic.

Just as in story one, the fate of the population overall seems very similar in these simulations of story two. However, the change in dataset this time has brought the impact of the seeding alteration and clustering process to the forefront at an individual level. Hence, these simulations highlight how somewhat innocent changes (to the algorithm) can have huge consequences for individuals even if the population as a whole seems to be unaffected.

This outcome connects back to the proposed vaccination programme in Chinatown, how would recipients have been decided? If the only criteria were being “Chinese”, how, for example, would a child with one Chinese parent and one white-American parent be viewed? While such parentage would have been condemned—probably by both races—there were almost certainly cases, due to the popularity of Chinese brothels frequented by white men (Light 1974). Would such children have been given the vaccine? Depending on which group individual children were clustered into, their fates could have varied wildly.

### Story three: Deviation from instruction—How an AI system could go rogue

The two preceding stories, from a population level viewpoint, are fairly similar and tame. On the contrary, story three’s simulations are far from tame, and the change of seed has equally as dramatic consequences as the change in dataset. Arguably, it is in this run of the toy model’s simulations that the ‘fear of the algorithm’ idea, that is popular in Hollywood, starts to appear more like reality. Although, once again it is noted that this model is idealised, this model has random and stochastic elements as previously mentioned that have made its outcomes a relative surprise even to the authors of this paper. Hence, this story undoubtedly demonstrates the reality of the negative outcomes that were supposedly disproven in story one and that were hinted at when this paper began.

This story’s simulations use both of the health parameters, age and weight.

**Fig. 15:**
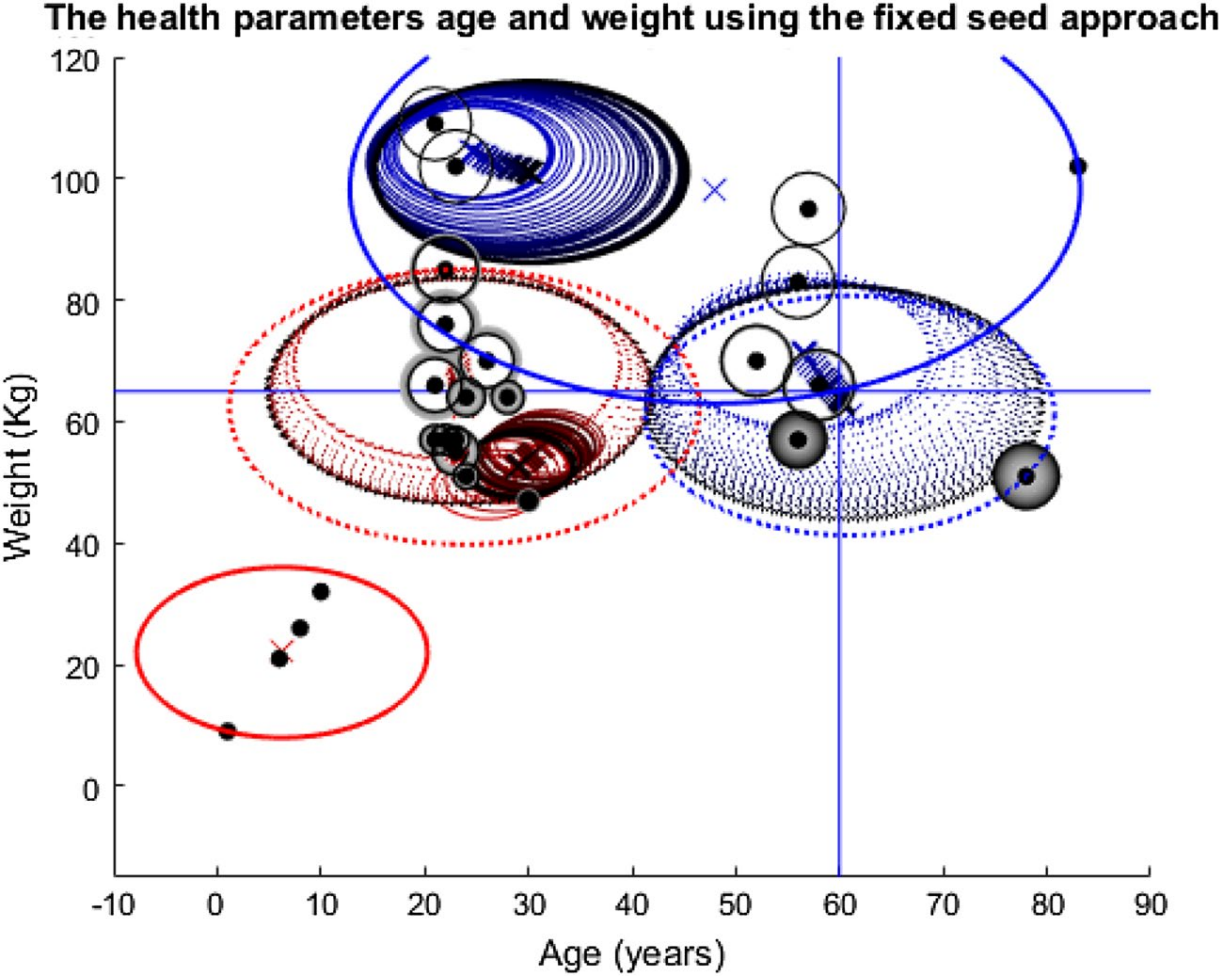
Plot depicting the population according to their ages and weights using the fixed seeding. The blue lines at y = 65 and x = 60 mark the thresholds for vaccination. For details on how to read the plot, see Fig. 8 caption

Evidently, Fig. 15 portrays very different results compared to the previous simulations of its kind. The bottom left red cluster is excluded from the very beginning. This forces the algorithm to seek its fourth cluster elsewhere, hence the small solid red (becoming black) cluster in the middle of the red-dotted one. The two dotted clusters almost recover to their initial size but the blue solid one does not which leaves the top right node excluded and unvaccinated.

**Fig. 16:**
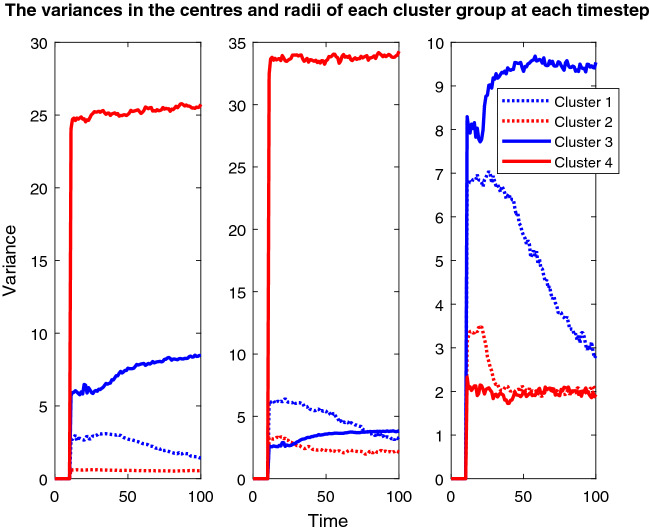
The left picture shows the variance in the x-value of the cluster centres over time, the centre picture shows the y-value and the right plot shows the variance in the radius of each cluster group at each timestep. This plot corresponds to the fixed seeding age vs weight plot depicted in Fig. 15 and its colours and dotted/solid line variations correspond to the cluster groups in that plot

Figure 16 mainly confirms the dramatic change seen in the solid red cluster group’s position in Fig. 15 and the dramatic size change in the blue solid cluster group. Hence, it helps to prove the unpredictable nature of this simulation and how the algorithm has deviated away from its starting position.

**Fig. 17:**
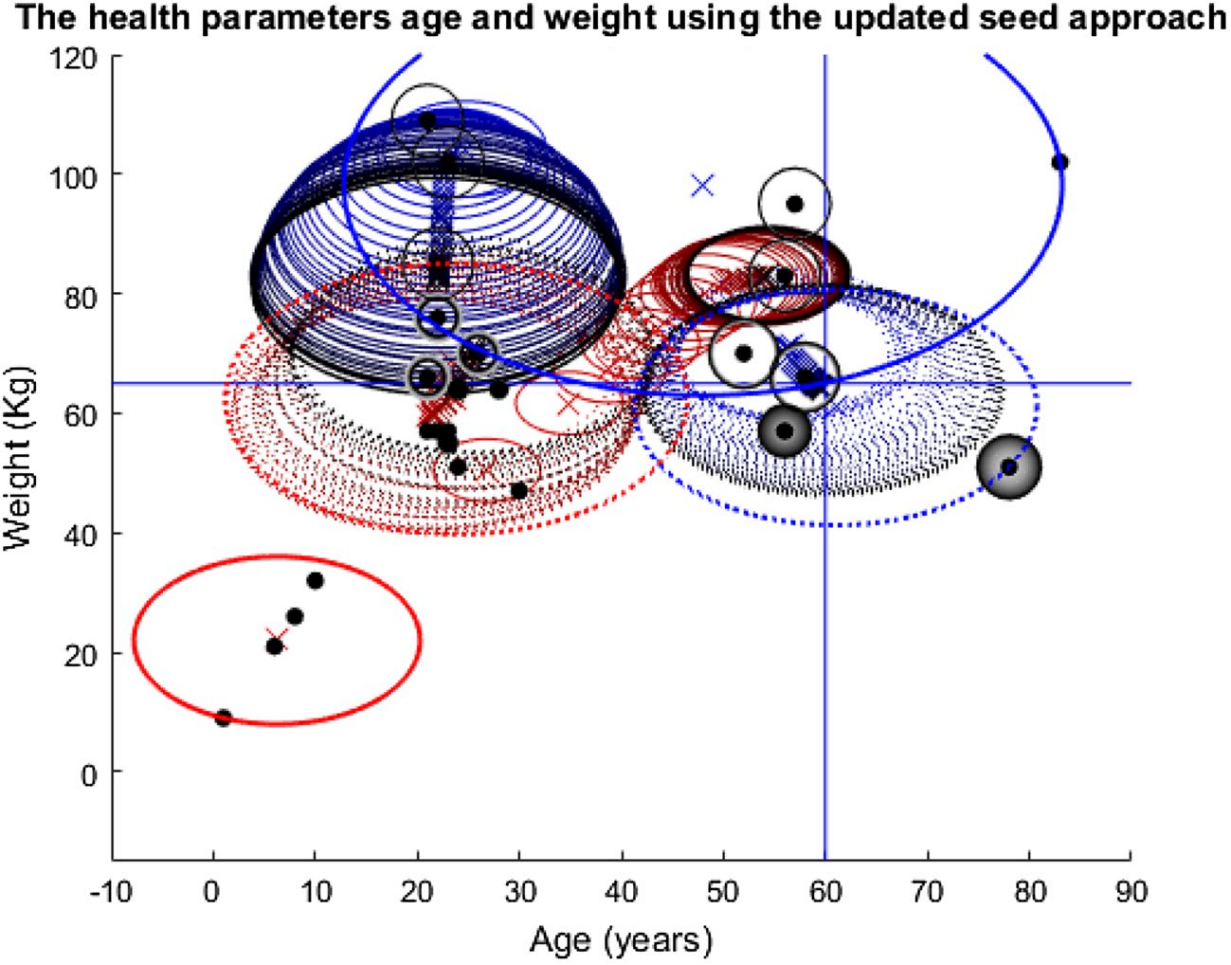
Plot depicting the population according to their ages and weights using the fixed seeding. The blue lines at y = 65 and x = 60 mark the thresholds for vaccination. For details on how to read the plot, see Fig. 8 caption

There are similarities between Figs. 15 and 17, such as the immediate exclusion of the bottom left cluster group, the exclusion of the top right node and the almost recovery of both the dotted clusters. However, contrary to the previous two stories’ population-wide similarities, that is where the common ground between the two seeding approaches for this pair of features ends. The red solid cluster group is again small in Fig. 17, but this time it is in a completely new location and moves much more. Similarly, the blue solid cluster this time both fails to recover to its previous size, and it has shifted over to the left significantly.

**Fig. 18:**
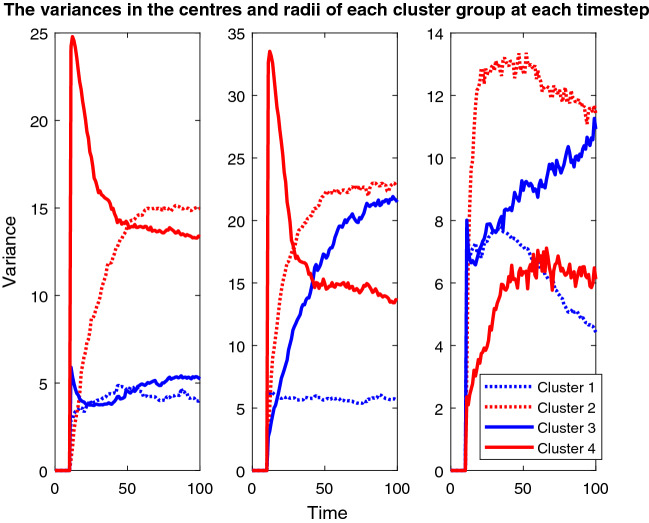
This plot corresponds to the updated seeding age vs weight plot depicted in Fig. 17 and its colours and dotted/solid line variations correspond to the cluster groups in that plot. For further detail on reading this plot, see the caption for Fig. 16

As with the relationship between Figs. 15 and 16, 18 mainly confirms the dramatic movement and changes in size for the cluster groups in Fig. 17. The changes in the position of the red solid line are very drastic in the first two plots in Fig. 18. Yet, all the other cluster groups vary a lot too. The differences between Figs. 16 and 18 are undeniable and dramatic. Therefore, story three’s results have highlighted the impact of the change in datasets, with Figs. 15 and 17 looking completely different to their corresponding plots in the other two stories. However, the differences between Figs. 16 and 18 have also highlighted the impact of the change in seeding on the outcomes for the population.

Story three’s simulations have shown that despite the initial model parameters being set, as shown in Table 1, and who was to be vaccinated being defined, the re-clustering and exclusion processes have altered the shape of this population to the extent that the algorithm seems to be diverting from its original instructions. However, it is the initial instruction to have exactly four cluster groups that has caused the variance in all of the cluster groups, once the bottom left group had been excluded, the algorithm must find its fourth group elsewhere. It could not have been foreseen that that entire cluster group would be constantly excluded, hence the outcomes of this simulation were equally impossible to predict. Herein lies the danger when programming AI systems, the outcomes caused by the innocent change in dataset and experimentation with the seed have demonstrated how certain programmers need to be in the results of their programmes before releasing them into the real world.^2^

## Discussion

The bias may have been written into all the simulations in this paper due to the fact that decisions over vaccinations are based on using weight as a feature in the dataset. The inputting of a biased dataset, rings comparable, if intrinsically different, alarm bells to those of San Francisco in 1900. Those health officials may have used health records to identify who ‘needed’ the vaccine. The programmer of the toy model above holds a similar role and uses records of age, weight and edge degree to determine vaccine receipt. In both these cases, the prejudice is written in, be it via a prejudiced dataset or prejudiced thoughts inherent to the time period. However, where this toy model deviates from the San Francisco case study is where bias emerges separate to this inherent feature. The further biases that emerged in both story two, against the individual, and in story three, against particularly the youngest cluster, could not have been predicted. In the simulations in story three, bias emerged on top of what was dictated, simply by the algorithm’s requirement to follow the initial instructions to the end, despite it no longer being the best option for the population. The feedback loop that emerged between clustering, exclusion and vaccination proved impossible in story three for the algorithm to escape from. Importantly, even if the initial prejudice that was written in here was completely unintended, the emergent bias would remain an outcome from the AI system. The idea of intended or unintended (emergent) bias is not distinguishable to an AI.

As previously stated, it is unlikely that such a simplistic model would be utilised in the vaccination context. However, similar such models are already in action; as an example, consider crime prediction software in America, specifically PredPol. This system looks at crime in one area and uses historical patterns to predict when and where it might occur next (O’Neill 2016). On the surface, this seems highly effective; the model is blind to race and ethnicity since it simply targets geography (O’Neill 2016). Unfortunately, this desire can be evaded, as Nixon’s notorious “dog-whistle” words in his Southern Strategy apparently showed (D’Souza 2018). As in this case, geography can be an accurate proxy for race because impoverished neighbourhoods are primarily occupied by ethnic minorities (O’Neill 2016). This becomes more critical when nuisance crimes are included in the model, which are prevalent in impoverished neighbourhoods (O’Neill 2016). As with the idealised vaccination model, a negative feedback loop might well be created: policing creates new data, justifying more policing (O’Neill 2016). Evidently, there is an important difference between the toy vaccination model and Predpol; the dataset Predpol utilised was not intentionally biased, but both examples prove that care needs to be taken when programming AI systems. If not, there is a real danger of unconscious biases being fed into them or emerging. Importantly, this is not a definite outcome; prejudice does not have to be inherent as in San Francisco’s Chinatown. As Leonhard (2016: 75) accurately states, the ‘technology is neither good nor bad; it simply is. We must—now and here—decide and agree which exact use is evil or not’.

Predpol is not the only example of unintentional biases guiding the decisions of AI systems. Examples of this phenomenon are commonplace. Even the big technology giants are not immune. In 2018, it came to light that Amazon had to scrap its AI recruiting tool because it was biased against women (Dastin 2018). Their model was trained on applications submitted to the company over the past ten years, which reflected the male dominance of the industry (Dastin 2018). The AI system was trained on the data of the past resumés, whilst the new resumés became the so-called test data. It was from the training data that the AI picked up the bias against women, it then transferred this knowledge onto the test data. Therefore, the system learnt that male candidates were preferable and penalised the resumés that included words such as ‘women’s’ and downgraded graduates of two all-female colleges (Dastin 2018). This obviously had not been Amazon’s intention but their lack of consideration of the potential consequences from using the biased dataset, resulted in a biased recruitment tool. By simply changing the dataset used, the outcome could have been very different for the women who were rejected purely based on their gender, since the system would not have learnt to exclude them. However, as the toy vaccination model proved, changing the dataset may well improve the outcomes for one individual or group but equally likely, it could cause other individuals or groups to be even worse off. If Amazon are having such difficulties, then it is unsurprising that others are too, with stories of ‘biased AI’ frequently in the news.

One upsetting example, that combines all the elements of this article; the prejudiced backdrop of decisions, the impact on individuals and the problems in clustering people into defined groups, is apartheid in South Africa. In the 1950s, those in power ‘sought to divide the population into four basic groups: Europeans, Asiatics, Persons of mixed race or [black people], and “natives” or “pure-blooded individuals of the Bantu race”’ (Bowker and Star 2000: 197). However, an entire population would never conform to such ‘simple’ groupings, making the process completely inconsistent (Bowker and Star 2000). Despite the problems, a person’s racial classification could be challenged at any time, even if you merely associated with someone of the ‘wrong group’ (Bowker and Star 2000). Also, these cluster groups were not just administrative, they determined where a person could live and work (Bowker and Star 2000). In effect this process ‘separated families, disrupted biographies, and damaged individuals beyond repair’ (Bowker and Star 2000: 218). While this may be an extreme example of the consequences of classification, it does demonstrate the dangers of trying to make individuals fit into groups. As Bowker and Star (2000: 224–225) state, ‘there can be tremendous torque of individual biographies’. The toy model in this paper demonstrates these damaging consequences for individuals from over-simplifying a population into a set number of groups. Particularly, its outcomes in stories two and three show how algorithms will not remove this potential for damaging ‘torques’ should care not be taken when determining datasets and making ‘innocent changes’.

Unfortunately, these problems with classifying individuals are already arising in the technological world and they are relatively unchecked. Bearing in mind these historic problems with classification, it seems unlikely that the introduction of AI will immediately fix the problems. Once again, choices regarding datasets are wreaking havoc in multiple areas. For example, Buolamwini (2016) first encountered exclusion problems with generic facial recognition software with social robots. The robot could not classify her face as such unless she wore a white mask (Buolamwini 2016). Such facial recognition software are often trained on similar datasets, but if these are not sufficiently diverse, then any face that deviates from what the AI has learnt to be a face will not be detected as such (Buolamwini 2016).

Not being classified at all by this kind of software is not the only problem. In 2015 Google’s Photos service labelled a black software developer and his friend as ‘gorillas’ and since then, their main solution has been deleting gorilla from the service’s options (Simonite 2018). These problems in facial recognition are awful for the people affected and embarrassing for the companies involved. However, the consequences become scarier still when considering some of the uses for these technologies. For example, Google are branching out into autonomous vehicles, which use image recognition software to classify the world around them. The potential consequences of that hypothetical scenario are best left to your imagination.

## Conclusion

Despite this paper’s simulations being idealised, the way it maps onto real-life examples of negative feedback loops is important to change how we manage these technologies. The model’s consequences may be exaggerated but the consequences detailed in the case studies above were very real. As already stated, it is extremely unlikely that such a simplistic modelling technique would be used in reality in healthcare. However, it has been used here to highlight a feedback loop between datasets and outcomes which are not idealised. Such feedback loops do exist and are already having negative impacts on certain groups’ lives as seen in the examples offered. All of the points raised by the toy model; feedback loops, dataset choices and small changes in code are all tied together and impacting on each other to have sometimes disastrous consequences. Clearly, people do not fit neatly into groups and this toy model has shown that by simply changing the dataset and seeding, the outcome for an individual can be completely different. The apparent human need to classify things, and in this case, people, is visible throughout our history (Bowker and Star 2000). Equally visible, however, are the problems that come with this desire to sort people and there will always be anomalies. If this is forgotten, then the true impact of oversimplifying these decisions over datasets and variables, such as the seed, cannot be truly appreciated and may not be prioritised. As AI branches out into more areas of life the focus needs to be on protecting those members of society who could potentially suffer at the hands of a poorly considered AI system. It is vital we take heed from the examples that have gone before and consider this ‘toy model’s’ worst case scenario outlook and work to avoid it.

There is a consensus that the original intentions of AI are generally benign, making the term “bias AI” a little misleading. No programme will ever start out biased, they are like children, they have to be taught and they learn what their parents or, in this case, programmers and data tell them. Buolamwini (2016) calls this the ‘Coded Gaze’, when the views that are embedded into systems are propagated by those who have the power to code the systems. ‘Whoever codes the system embeds her [their] views. Limited views create limited systems’ (Buolamwini 2016). As demonstrated in the many examples in this article, this embedding could be intentional or unconscious, but the consequences are the same. These consequences not only impact groups of our society as a whole but also can have nuanced impacts on individuals within the population.

There is much more scope for further work in this area that can build on the simple stochastic network models used in this paper. Alternative approaches/more complex examples have been noted in the introduction. These could be used to develop the many ways in which this paper’s work, models and conclusions could be extended and taken more into ‘reality’. Models in this paper have focussed on the impact AI could have on healthcare today, one of the most current and certainly topical dilemmas in the field. A report from the NHS in the UK recognises the problems with data as discussed in this research but equally, they recognise the opportunity that AI presents for improvement and therefore to save lives (Harwich and Laycock 2018). But to blame AI and present it as ‘bad’ is to paint too simple a picture, the bias that is appearing in systems in all areas is often inherited from the data or even the programmer, whether it is deliberate or unconscious is another question. Equally, learning from the past will prevent making the same mistakes again. Unless care is taken, prejudices such as those that governed both San Francisco’s Chinatown in 1900 and South Africa under apartheid will continue to emerge, only now they will emerge through AI systems. Stopping and considering the datasets we are using, what the consequences of a seemingly harmless decision could be, will enable us to get the best out of this bright, new technology.

## Supplementary Information

Below is the link to the electronic supplementary material.

Supplementary file1 (DOCX 37 KB)

## Footnotes

1 In fact, age is one of the nine protected characteristics that it is against the law to discriminate against (Equality and Human Rights Commission 2020). That is not to say that determining distribution of flu vaccines by age is unlawful since ‘a service provider is [essentially] making age-related concessions and benefits’ (Equality and Human Rights Commission 2020). On the other hand, weight is not one of the nine protected characteristics, but to use it to determine a vaccination programme would more than likely be deemed unethical.

2 One real-world example of this was the A-level results fiasco in Britain during the Summer of 2020. Due to the COVID-19 outbreak, A-level exams were cancelled. Instead student’s results were based on an algorithm that used each school’s past performance as a parameter (BBC News 2020). However, the reliance this algorithm had on this parameter meant that some high-achieving students at underperforming schools had their results downgraded (BBC News 2020). On the opposite side, this meant that the results for private schools were benefitted (BBC News 2020). This is just one example of the potential negative outcomes should the parameters inputted into algorithms be poorly considered and then not noticed until it is too late.
