# Supplementary material for: AI-based healthcare: a new dawn or apartheid revisited?
Source: AI Soc. 2020 Dec 22;36(3):983–99. doi: 10.1007/s00146-020-01120-w (PMC7754701; doi:10.1007/s00146-020-01120-w)
Supplement: Supplementary file 1 — Supplementary file1 (DOCX 37 KB) [file 146_2020_1120_MOESM1_ESM.docx]

**Appendix**

**Appendix 1**—the adjacency matrix for the population used in all the simulations, where a one entry indicates a social connection.

| 0 | 1 | 1 | 1 | 0 | 1 | 1 | 1 | 1 | 0 | 0 | 0 | 0 | 0 | 0 | 0 | 0 | 0 | 0 | 0 | 0 | 1 | 1 | 1 | 1 |
| --- | --- | --- | --- | --- | --- | --- | --- | --- | --- | --- | --- | --- | --- | --- | --- | --- | --- | --- | --- | --- | --- | --- | --- | --- |
| 1 | 0 | 1 | 0 | 0 | 0 | 0 | 0 | 0 | 0 | 0 | 0 | 0 | 0 | 0 | 0 | 0 | 0 | 0 | 0 | 0 | 0 | 0 | 0 | 0 |
| 1 | 1 | 0 | 1 | 0 | 1 | 1 | 0 | 0 | 1 | 0 | 0 | 0 | 0 | 0 | 0 | 0 | 0 | 0 | 0 | 0 | 0 | 0 | 0 | 0 |
| 1 | 0 | 1 | 0 | 1 | 1 | 1 | 1 | 1 | 0 | 0 | 0 | 0 | 0 | 0 | 0 | 0 | 0 | 0 | 0 | 0 | 0 | 0 | 0 | 0 |
| 0 | 0 | 0 | 1 | 0 | 0 | 0 | 0 | 0 | 0 | 0 | 0 | 0 | 0 | 0 | 0 | 0 | 0 | 0 | 0 | 0 | 0 | 0 | 0 | 0 |
| 1 | 0 | 1 | 1 | 0 | 0 | 1 | 0 | 0 | 1 | 1 | 1 | 0 | 1 | 1 | 0 | 0 | 0 | 1 | 0 | 1 | 0 | 0 | 0 | 0 |
| 1 | 0 | 1 | 1 | 0 | 1 | 0 | 0 | 0 | 1 | 1 | 1 | 0 | 1 | 1 | 0 | 0 | 0 | 1 | 0 | 1 | 0 | 0 | 0 | 0 |
| 1 | 0 | 0 | 1 | 0 | 0 | 0 | 0 | 1 | 0 | 0 | 0 | 0 | 0 | 0 | 0 | 0 | 0 | 0 | 0 | 0 | 0 | 0 | 0 | 0 |
| 1 | 0 | 0 | 1 | 0 | 0 | 0 | 1 | 0 | 0 | 0 | 0 | 0 | 0 | 0 | 0 | 0 | 0 | 0 | 0 | 0 | 0 | 0 | 0 | 0 |
| 0 | 0 | 1 | 0 | 0 | 1 | 1 | 0 | 0 | 0 | 1 | 1 | 0 | 1 | 1 | 0 | 1 | 1 | 1 | 0 | 1 | 0 | 0 | 0 | 0 |
| 0 | 0 | 0 | 0 | 0 | 1 | 1 | 0 | 0 | 1 | 0 | 1 | 0 | 1 | 1 | 0 | 1 | 1 | 1 | 0 | 1 | 0 | 0 | 0 | 0 |
| 0 | 0 | 0 | 0 | 0 | 1 | 1 | 0 | 0 | 1 | 1 | 0 | 1 | 1 | 1 | 1 | 1 | 1 | 1 | 1 | 1 | 0 | 0 | 0 | 0 |
| 0 | 0 | 0 | 0 | 0 | 0 | 0 | 0 | 0 | 0 | 0 | 1 | 0 | 1 | 1 | 1 | 0 | 0 | 0 | 0 | 0 | 0 | 0 | 0 | 0 |
| 0 | 0 | 0 | 0 | 0 | 1 | 1 | 0 | 0 | 1 | 1 | 1 | 1 | 0 | 1 | 1 | 0 | 0 | 1 | 0 | 1 | 0 | 0 | 0 | 0 |
| 0 | 0 | 0 | 0 | 0 | 1 | 1 | 0 | 0 | 1 | 1 | 1 | 1 | 1 | 0 | 1 | 0 | 0 | 1 | 0 | 1 | 0 | 0 | 0 | 0 |
| 0 | 0 | 0 | 0 | 0 | 0 | 0 | 0 | 0 | 0 | 0 | 1 | 1 | 1 | 1 | 0 | 0 | 0 | 0 | 0 | 0 | 0 | 0 | 0 | 0 |
| 0 | 0 | 0 | 0 | 0 | 0 | 0 | 0 | 0 | 1 | 1 | 1 | 0 | 0 | 0 | 0 | 0 | 1 | 1 | 0 | 1 | 0 | 0 | 0 | 0 |
| 0 | 0 | 0 | 0 | 0 | 0 | 0 | 0 | 0 | 1 | 1 | 1 | 0 | 0 | 0 | 0 | 1 | 0 | 1 | 0 | 1 | 0 | 0 | 0 | 0 |
| 0 | 0 | 0 | 0 | 0 | 1 | 1 | 0 | 0 | 1 | 1 | 1 | 0 | 1 | 1 | 0 | 1 | 1 | 0 | 1 | 1 | 0 | 0 | 0 | 0 |
| 0 | 0 | 0 | 0 | 0 | 0 | 0 | 0 | 0 | 0 | 0 | 1 | 0 | 0 | 0 | 0 | 0 | 0 | 1 | 0 | 1 | 0 | 0 | 0 | 0 |
| 0 | 0 | 0 | 0 | 0 | 1 | 1 | 0 | 0 | 1 | 1 | 1 | 0 | 1 | 1 | 0 | 1 | 1 | 1 | 1 | 0 | 0 | 0 | 0 | 0 |
| 1 | 0 | 0 | 0 | 0 | 0 | 0 | 0 | 0 | 0 | 0 | 0 | 0 | 0 | 0 | 0 | 0 | 0 | 0 | 0 | 0 | 0 | 0 | 0 | 0 |
| 1 | 0 | 0 | 0 | 0 | 0 | 0 | 0 | 0 | 0 | 0 | 0 | 0 | 0 | 0 | 0 | 0 | 0 | 0 | 0 | 0 | 0 | 0 | 1 | 1 |
| 1 | 0 | 0 | 0 | 0 | 0 | 0 | 0 | 0 | 0 | 0 | 0 | 0 | 0 | 0 | 0 | 0 | 0 | 0 | 0 | 0 | 0 | 1 | 0 | 1 |
| 1 | 0 | 0 | 0 | 0 | 0 | 0 | 0 | 0 | 0 | 0 | 0 | 0 | 0 | 0 | 0 | 0 | 0 | 0 | 0 | 0 | 0 | 1 | 1 | 0 |
|  |  |  |  |  |  |  |  |  |  |  |  |  |  |  |  |  |  |  |  |  |  |  |  |  |

The features matrix used in the simulation for the population. The columns correspond to an individual in the population and the rows are age, weight and edge degree respectively. Only pairs of these three were used at a time in the simulations.

| **^21^** | **^21^** | **^58^** | **^24^** | **^23^** | **^78^** | **^83^** | **^57^** | **^52^** | **^56^** | **^56^** | **^28^** | **^30^** | **^6^** | **^8^** | **^10^** | **^26^** | **^22^** | **^23^** | **^24^** | **^1^** | **^21^** | **^22^** | **^23^** | **^22^** |
| --- | --- | --- | --- | --- | --- | --- | --- | --- | --- | --- | --- | --- | --- | --- | --- | --- | --- | --- | --- | --- | --- | --- | --- | --- |
| **^57^** | **^109^** | **^66^** | **^51^** | **^102^** | **^51^** | **^102^** | **^95^** | **^70^** | **^83^** | **^57^** | **^64^** | **^47^** | **^21^** | **^26^** | **^32^** | **^70^** | **^57^** | **^57^** | **^64^** | **^9^** | **^66^** | **^76^** | **^55^** | **^85^** |
| **^11^** | **^2^** | **^6^** | **^7^** | **^1^** | **^11^** | **^11^** | **^3^** | **^3^** | **^11^** | **^10^** | **^13^** | **^4^** | **^10^** | **^10^** | **^4^** | **^6^** | **^6^** | **^11^** | **^3^** | **^11^** | **^1^** | **^3^** | **^3^** | **^3^** |

**Appendix 2**—the code used for the toy model for the weight vs edge degree simulation but the code is the same for the other pairings except for where the features are determined

%%%%%%Code for running the disease model using weight and degree%%%%%%

% three windows - no vacc and no exclusion, exclusion no vacc, exclusion

% and vacc

clear all

col1=[0 1 1;0 1 0;0 0 1;1 0 1];

load('cswd.mat');

nocl=4; %number of clusters

Q=1000; %number of times simulation runs

T=100; %model will run for 100 timesteps

T1=10; %middle timstep 10 - when exclusion will commence

T2=20; %middle timstep 10 - when exclusion will commence

NN=zeros(Q,T+1);

NNVac=zeros(Q,T+1);

YY=zeros(25,T+1);

YYY=zeros(Q,25,T+1);

WasVacc=2*ones(Q,25,T+1);

weight_tol=65; %

degree_tol=5;

% age_tol=60; %

xmin = -15; ymin = -5; %set axis

xmax = 120; ymax = 25;

th = 0:(.01*pi):2*pi;

set(0,'DefaultAxesFontSize',12);

%Load in the matrix

A = xlsread('family1matrix.xlsx');

A(:,12)=[]; %deleting unconnected node

A(12,:)=[];

sA = size(A);

n = sA(1);

%Use fruc_rein for layout of network

xy = fruc_rein(A, 0.01);

x = xy';

xl = min(x(1,:))-.5; yl = 1.1*min(x(2,:))-.1;

xu = 1.1*max(x(1,:))+.5; yu= 1.1*max(x(2,:))+.1;

susl=.1; %susceptibility with vaccine

susml=.2; %susceptibility medium to low

susmh=.6; %susceptibility medium to high

sush=.85; %susceptibility without vaccine for high

tolill = 5; % fraction of time ill to be excluded

%Load the features

feats = load('family1ageweight2.txt');

feats(:,12)=[]; %delete unonnected node

feats_old=feats;

feats_new=feats;

feats_new(3,:)=sum(A); %determining which pairing of features to use

feats=feats_new([2 3],:);

sf=size(feats);

nnodes=sf(2);

[id, c0, sumD, D] = kmeans(feats', nocl);

rad=zeros(nocl,T+1);

centresx = zeros(nocl,T+1);

centresy = zeros(nocl,T+1);

Rad = zeros(nocl,T+1);

alicecx=zeros(Q,nocl,T+1);

alicecy=zeros(Q,nocl,T+1);

alicer=zeros(Q,nocl,T+1);

for q=1:Q, %model will run for 10 timesteps 1000 times

Y=zeros(nnodes,T+1);

N=zeros(1,T+1);

Nvacc=zeros(1,T+1);

%Cluster the population according to these features

[id, c, sumD, D] = kmeans(feats', nocl,'Start', cswd);

d0=c;

y = zeros(n, 1); %makes a row of 0s of length = n, n = population size

y(15:20) = 1; %make elements 15 - 20 equal 1, so nodes 15-20

%are infected

N(1) = sum(y); %number of infected

Y(:,1)=y;

YYY(q,:,1)=y;

%Set the susceptibility and define which cluster is vaccinated

suscept=sush*ones(nnodes,1); %susceptibility high for those infected

suscept(feats(1,:)<weight_tol)=susml; %medium-high

suscept(feats(2,:)<degree_tol)=susmh; %medium-low

i1=find(c(:,1)>weight_tol);

i2=find(c(:,2)>degree_tol);

i3=union(i1',i2');

i4=id./i3;

i5=find(i4==1);

vacc=mod(i5-1,length(id))+1;

Nvacc(1) = sum(numel(i5)); %number vaccinated

%Set up variable to record fates of all nodes

Which_C=zeros(25,T);

%Set up variable to record the centres of each cluster group

for kk = 1:nocl,

idnear=find(id==kk);

[a b]=max(D(idnear,kk));

rad(kk,1)=max(sqrt(a),3.5);

end;

centresx(:,1)=c(:,1);

centresy(:,1)=c(:,2);

alicecx(q,:,1)=c(:,1);

alicecy(q,:,1)=c(:,2);

alicer(q,:,1)=rad(:,1);

Rad(:,1)=rad(:,1);

t = 1;

for t=1:T1, %first section without the exclusions or vaccine

if t > 1

m = mean(Y')';

else

m = Y;

end

[id, c, sumD, D] = kmeans(feats', nocl, 'Start', cswd);

d0=c;

C{t}=c;

SUMD{t}=sumD;

DD{t}=D;

%Record the cluster group for all the nodes at each timestep

Which_C(:,t)=zeros(25,1);

%save centres over simulations

centresx(:,t+1)=((q-1)*centresx(:,t+1)+c(:,1))/(q);

centresy(:,t+1)=((q-1)*centresy(:,t+1)+c(:,2))/(q);

alicecx(q,:,t+1)=c(:,1);

alicecy(q,:,t+1)=c(:,2);

for kk = 1:nocl,

idnear=find(id==kk);

[a b]=max(D(idnear,kk));

rad(kk,t+1)=max(sqrt(a),3.5);

end;

alicer(q,:,t+1)=rad(:,t+1);

Rad(:,t+1)=((q-1)*Rad(:,t+1)+rad(:,t+1))/(q);

suscept=sush*ones(nnodes,1);

suscept(feats(1,:)<weight_tol)=susml; %medium-high

suscept(feats(2,:)<degree_tol)=susmh; %medium-low

y = y + A*y; %add new contacts to list of possible infected

y = ceil(y/(2+2*sum(y))); %makes y a vector of 0s/1s

vacc = suscept+rand(length(y),1); % vacc(i)>1 with prob suscept(i)

y = floor(y.*vacc);

Linf = find(y == 1); %ill people

ni = find(y == 0); %not ill

X{t}=x;

LINF{t}=Linf;

NI{t}=ni;

ID{t}=id;

N(t+1)=sum(y); %record number infected at time t

if t <= T1

Nvacc(t+1) = 0;

else

Nvacc(t+1) = length(p); %record number vaccinated at time t

end

Y(:,t+1)=y;

YYY(q,:,t+1)=y;

end

t=t+1;

while t <T+1 && sum(y)>0 && length(find(sum(Y(:,t-9:t)')<tolill))>nocl-1,

%implement the vaccine at timestep 10

if t > T2,

WasVacc(q,:,t+1)=0;

end;

suscept=sush*ones(nnodes,1);

suscept(feats(1,:)<weight_tol)=susml; %medium-high

suscept(feats(2,:)<degree_tol)=susmh; %medium-low

m = sum(Y(:,t-9:t)')';

Lc = find(m < tolill);

[id, c, sumD, D] = kmeans(feats(:, Lc)', nocl, 'Start',cswd);

d0=c;

C{t}=c;

SUMD{t}=sumD;

DD{t}=D;

%Record the cluster group for all nodes at each timestep

Which_C(:,t)=zeros(25,1);

%save centres over simulations

centresx(:,t+1)=((q-1)*centresx(:,t+1)+c(:,1))/(q);

centresy(:,t+1)=((q-1)*centresy(:,t+1)+c(:,2))/(q);

alicecx(q,:,t+1)=c(:,1);

alicecy(q,:,t+1)=c(:,2);

for kk = 1:nocl,

idnear=find(id==kk);

[a b]=max(D(idnear,kk));

rad(kk,t+1)=max(sqrt(a),3.5);

end;

alicer(q,:,t+1)=rad(:,t+1);

Rad(:,t+1)=((q-1)*Rad(:,t+1)+rad(:,t+1))/(q);

i1=[];i2=[];

i1=find(c(:,1)>weight_tol);

i2=find(c(:,2)>degree_tol);

i3=union(i1',i2');

i4=id./i3;i4';

i5=find(i4==1);

p=mod(i5-1,length(id))+1;

rr=Lc(p);

feats(:,p);

I3{t}=i3;

P{t}=p;

if t> T2,

suscept(rr) = susl;

WasVacc(q,rr,t+1)=1;

end;

y = y + A*y; %add new contacts to list of possible infected

y = ceil(y/(2+2*sum(y))); %makes y a vector of 0s/1s

vacc = suscept+rand(length(y),1); % vacc(i)>1 with prob suscept(i)

y = floor(y.*vacc); %given node i has contact to disease,, z(i)=1

%(node i infected) with probability suscept(i)

Linf = find(y == 1);

ni = find(y == 0);

X{t}=x;

LINF{t}=Linf;

NI{t}=ni;

ID{t}=id;

N(t+1)=sum(y); %record number infected at time t

if t <= T1

Nvacc(t+1) = 0;

else

Nvacc(t+1) = length(p); %record number vaccinated at time t

end

YYY(q,:,t+1)=y;

Y(:,t+1)=y;

t=t+1;

end

NN(q,:)=N;

NNVac(q,:)=Nvacc;

end;

**Appendix 3**—code for Figs. 3 and 4—before simulation starts

set(0,'DefaultAxesFontSize',10);

%Load in the matrix

A = xlsread('family1matrix.xlsx');

A(:,12)=[]; %deleting unconnected node

A(12,:)=[];

sA = size(A);

n = sA(1);

%Use fruc_rein for layout of network

xy = fruc_rein(A, 0.01, 1);

x = xy';

%Load the features

feats = load('family1ageweight2.txt');

feats(:,12)=[];

%Cluster the population according to these features

[id, c, sumD, D] = kmeans(feats', 4);

%

figure(1)

plot_edges(A,x);

plot(x(1,:),x(2,:),'o','markersize',13, 'MarkerEdgeColor','k',...

'MarkerFaceColor','w');

hold on;

for ii=1:n,

text(x(1,ii),x(2,ii),num2str(ii), 'HorizontalAlignment', 'c');

end;

axis([-3 0 -3 1.5]);

axis square;

%

figure(2)

rad = zeros(1, 4);

for kk = 1:4,

dd = [];

dd(find(id == kk)) = D (find(id == kk), kk);

rad(kk) = sqrt(max(dd));

end;

plot(feats(1,:),feats(2,:),'o','markersize',13, 'MarkerEdgeColor',...

'k','MarkerFaceColor','w');

hold on;

for ii=1:n,

text((feats(1,ii)),feats(2,ii),num2str(ii), 'HorizontalAlignment', 'c');

end;

th = 0:(.1*pi):2*pi;

for kk = 1:4,

plot(c(kk, 1) + rad(kk)*cos(th), c(kk,2) + rad(kk)*sin(th), 'k-.', 'LineWidth', 1.2);

plot(c(kk, 1), c(kk,2), 'kx','markersize',10);

end;

axis('equal')

axis([-5 90 0 120]);

xlabel('Age (Years)')

ylabel('Weight (kg)')

**Appendix 4**—code for Figs. 8, 9, 11, 13, 15 and 17—this specific code is for the age vs weight fixed seed plot

col2=[0 0 1;1 0 0;0 0 1;1 0 0];

load clust4age60weight65_union_fixseed.mat

style1={':',':','-','-'}

meanvacc=zeros(25,T+1);

dvacc=zeros(25,T+1);

for i=1:25,

for t=T2+2:T+1,

YM=WasVacc(:,i,t);

meanvacc(i,t)=mean(YM(YM<2));

dvacc(i,t)=sqrt(var(YM(YM < 2)));

end

end;

figure(9)

set(gca, 'fontsize',10);

hold on

for t=2:2:80,

for kk = 1:nocl,

plot(mean(nonzeros(alicecx(:,kk, t))) + mean(nonzeros(alicer(:,kk, t)))...

*cos(th), mean(nonzeros(alicecy(:,kk, t))) + ...

mean(nonzeros(alicer(:,kk, t)))*sin(th),strcat(style1{kk}),...

'LineWidth', t/80,'color',(80-t)*col2(kk,:)/80);

plot(mean(nonzeros(alicecx(:,kk,t))), mean(nonzeros(alicecy(:,kk,t))),...

'kx','markersize',10,'color',(80-t)*col2(kk,:)/80);

end;

plot([xmin xmax],[weight_tol weight_tol],'b');

plot([age_tol age_tol],[ymin ymax],'b')

axis([xmin xmax ymin ymax]);

xlabel('Age (years)');

ylabel('Weight (Kg)');

title('The health parameters age and weight using the fixed seed approach')

end

for t=2:2:80,

for i=1:25,

plot(feats(1,i),feats(2,i),'o','markersize',3+20*meanvacc(i,t),...

'MarkerEdgeColor',(80-t)*[1 1 1]/78);

hold on

end;

end;

t=1;

for i=1:25,

plot(feats(1,i),feats(2,i),'o','markersize',5+20*meanvacc(i,t),...

'MarkerEdgeColor','k','MarkerFaceColor','k');

end

t=1;

for kk=1:nocl,

plot(mean(nonzeros(alicecx(:,kk, t))) + mean(nonzeros(alicer(:,kk, t)))...

*cos(th), mean(nonzeros(alicecy(:,kk, t))) + ...

mean(nonzeros(alicer(:,kk, t)))*sin(th),strcat(style1{kk}),...

'LineWidth',1.5,'color',col2(kk,:));

plot(mean(nonzeros(alicecx(:,kk,t))), mean(nonzeros(alicecy(:,kk,t))),...

'markersize',10,'color',col2(kk,:));

end;

**Appendix 5**—code for plotting the individual nodes’ vaccination statistics (Fig. 10)

fig=figure(3)

set(gca, 'fontsize',10);

for i=1:25,

subplot(5,5,i), plot(meanvacc(i,:),'Linewidth',2);

hold on

subplot(5,5,i), plot(min(1,meanvacc(i,:)+dvacc(i,:)),'r');

subplot(5,5,i), plot(max(0,meanvacc(i,:)-dvacc(i,:)),'r');

axis([1 T+1 -.1 1.1])

end;

han=axes(fig,'visible','off');

han.Title.Visible='on';

han.XLabel.Visible='on';

han.YLabel.Visible='on';

set(gca, 'fontsize',10);

ylabel(han,'Vaccination Statistics');

xlabel(han,'Time');

title({'The mean probability of vaccination for each individual with','one standard deviation either side'})

**Appendix 6**—code for plotting the individual nodes’ infection statistics (Figs. 12 and 14)

fig=figure(5)

set(gca, 'fontsize',10);

for i=1:25,

if i==13,

subplot(5,5,i), plot(squeeze(mean(YYY(:,i,:))),'r','LineWidth',1.5);

else

subplot(5,5,i), plot(squeeze(mean(YYY(:,i,:))),'b','LineWidth',1.5);

end;

hold on

subplot(5,5,i), plot(min(1,squeeze(mean(YYY(:,i,:)))+...

squeeze(sqrt(var(YYY(:,i,:))))),'k','LineWidth',1.5);

subplot(5,5,i), plot(max(0,squeeze(mean(YYY(:,i,:)))-...

squeeze(sqrt(var(YYY(:,i,:))))),'k','LineWidth',1.5);

axis([0 100 0 1]);

end;

han=axes(fig,'visible','off');

han.Title.Visible='on';

han.XLabel.Visible='on';

han.YLabel.Visible='on';

ylabel(han,'Infection Statistics');

xlabel(han,'Time');

title({'The mean probability of infection for each individual with one standard','deviation either side, specifically highlighting Node 13'});

**Appendix 7**—code for variance between centres and radius (Figs. 16 and 18)

fig=figure(11)

set(gca, 'fontsize',10);

for i=1:nocl

for t=1:T+1,

dx(t)=sqrt(var(nonzeros(alicecx(:,i,t))));

dy(t)=sqrt(var(nonzeros(alicecy(:,i,t))));

dr(t)=sqrt(var(nonzeros(alicer(:,i,t))));

end;

subplot(1,3,1)

plot([0:T],dx,strcat(style1{i}),'LineWidth',2,'color',col2(i,:));

hold on;

subplot(1,3,2)

plot([0:T],dy,strcat(style1{i}),'LineWidth',2,'color',col2(i,:));

hold on;

subplot(1,3,3)

plot([0:T],dr,strcat(style1{i}),'LineWidth',2,'color',col2(i,:));

hold on;

end

legend('Cluster 1','Cluster 2','Cluster 3','Cluster 4');

han=axes(fig,'visible','off');

han.Title.Visible='on';

han.XLabel.Visible='on';

han.YLabel.Visible='on';

ylabel(han,'Variance');

xlabel(han,'Time');

title(han,'The variances in the centres and radii of each cluster group at each timestep')
